# Supplementary figures and images for: Differential paralog divergence modulates genome evolution across yeast species
Source: PLoS Genet. 2017 Feb 14;13(2):e1006585. doi: 10.1371/journal.pgen.1006585 (PMC5308817; doi:10.1371/journal.pgen.1006585)

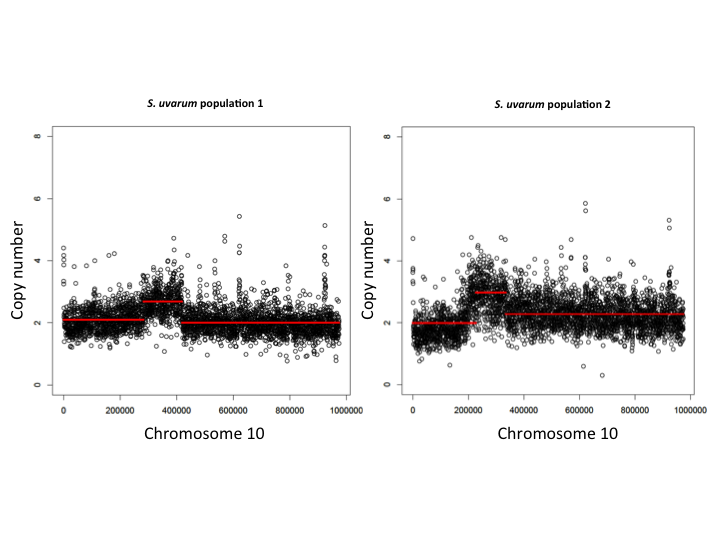

Supplement: S1 Fig — (TIFF) [file pgen.1006585.s004.tiff]

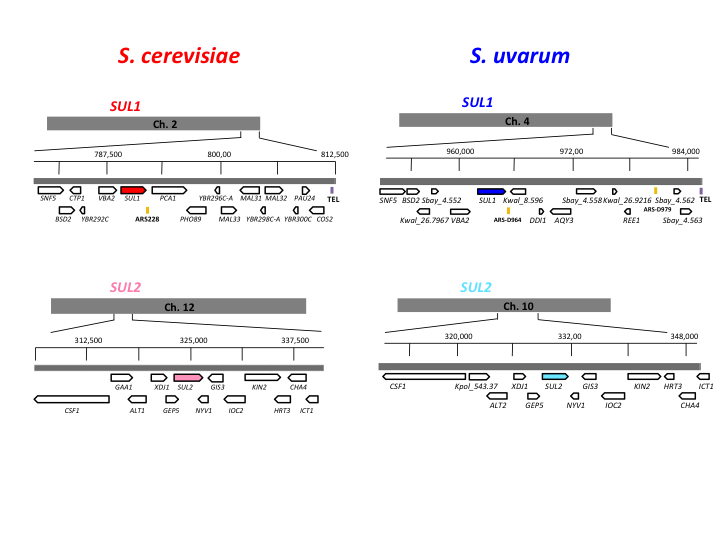

Supplement: S2 Fig — (TIFF) [file pgen.1006585.s005.tiff]

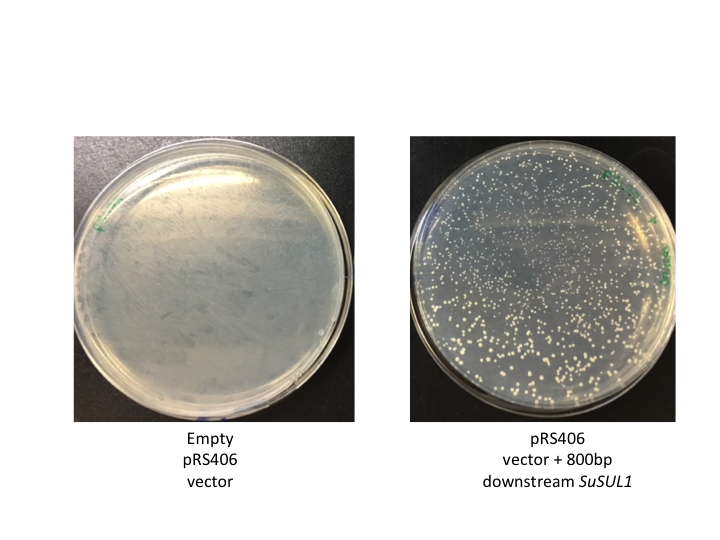

Supplement: S3 Fig — (TIFF) [file pgen.1006585.s006.tiff]

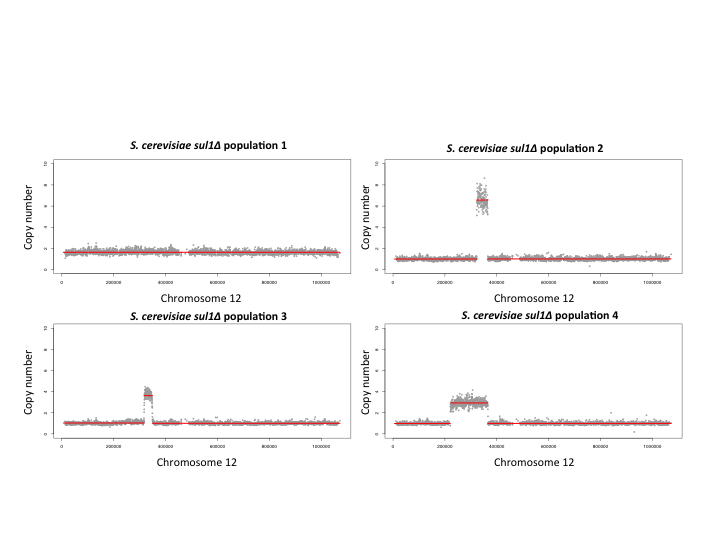

Supplement: S4 Fig — (TIFF) [file pgen.1006585.s007.tiff]

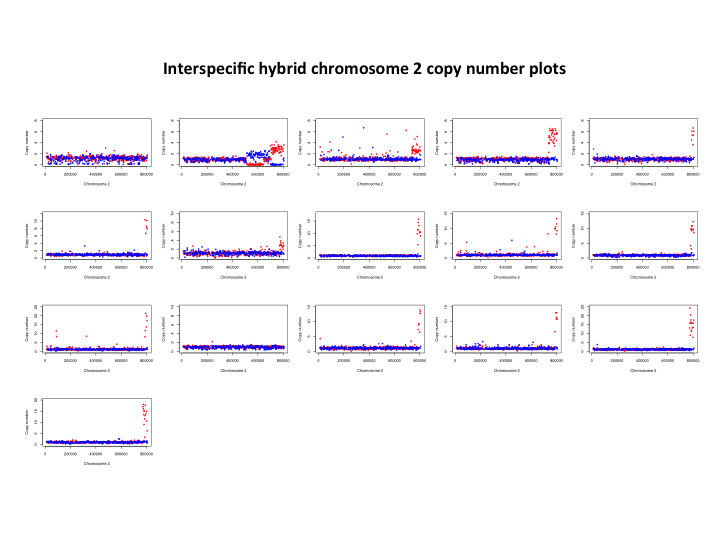

Supplement: S5 Fig — Note that each panel is scaled according to the range of values for that individual experiment. (TIFF) [file pgen.1006585.s008.tiff]

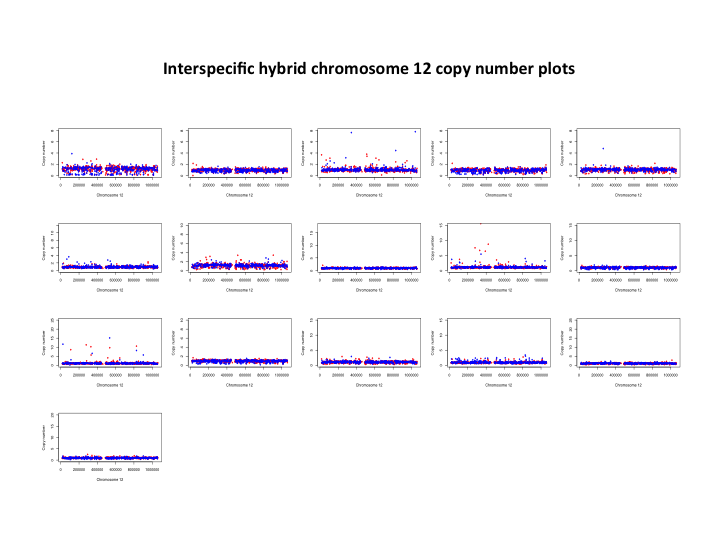

Supplement: S6 Fig — Note that each panel is scaled according to the range of values for that individual experiment. (TIFF) [file pgen.1006585.s009.tiff]

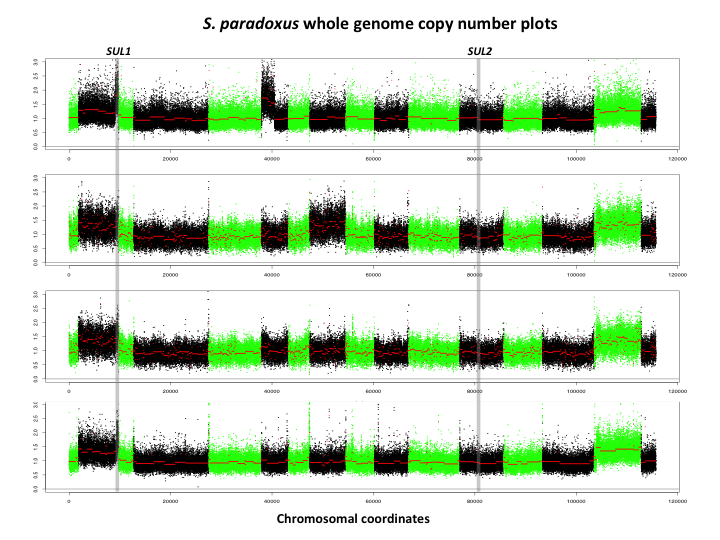

Supplement: S7 Fig — (TIFF) [file pgen.1006585.s010.tiff]

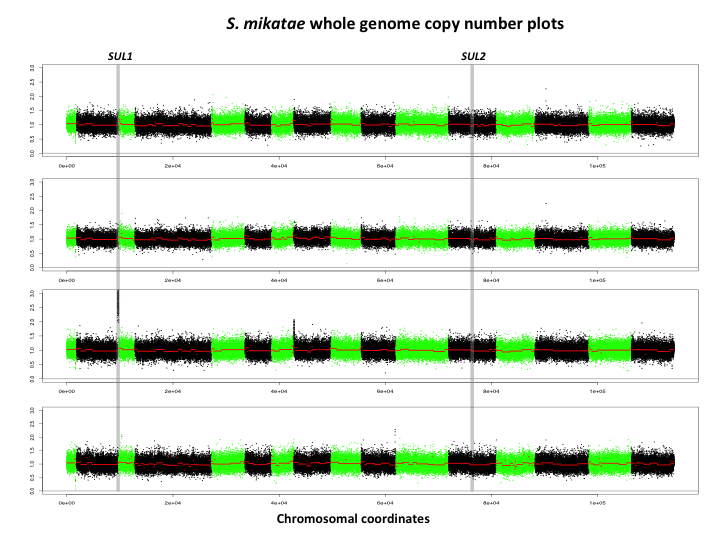

Supplement: S8 Fig — (TIFF) [file pgen.1006585.s011.tiff]

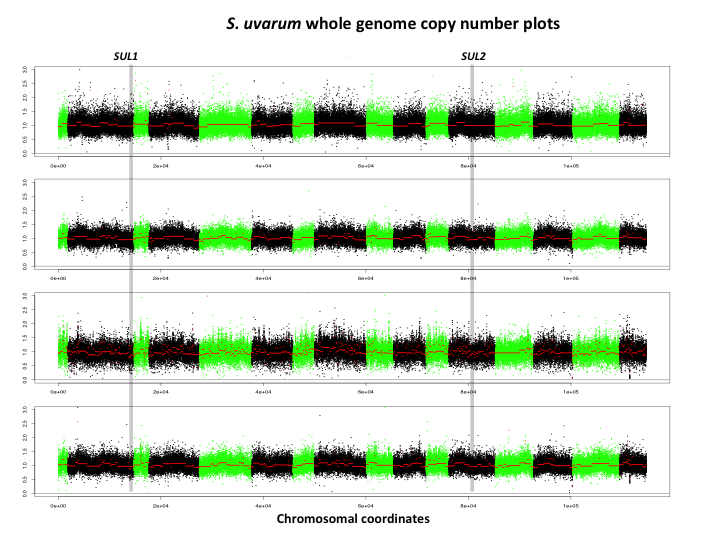

Supplement: S9 Fig — (TIFF) [file pgen.1006585.s012.tiff]

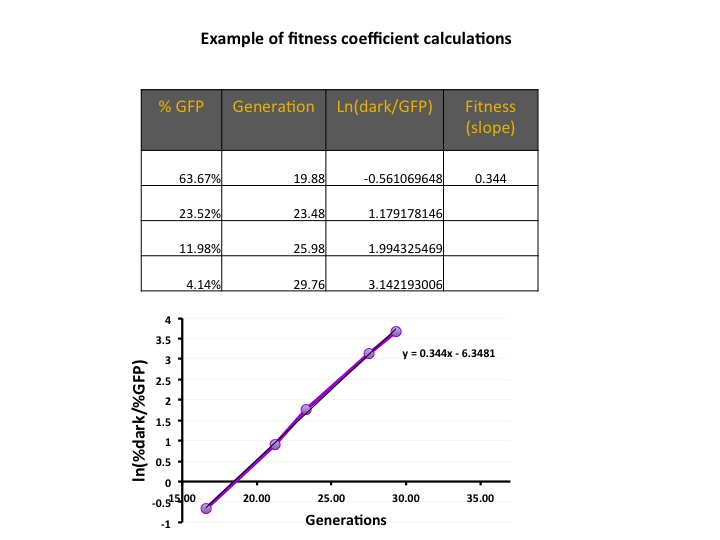

Supplement: S10 Fig — (TIFF) [file pgen.1006585.s013.tiff]
